# Squamous Cell Carcinomas of the Head and Neck Cancer Response to Programmed Cell Death Protein-1 Targeting and Differential Expression of Immunological Markers: A Case Report

**DOI:** 10.3389/fimmu.2018.01769

**Published:** 2018-07-30

**Authors:** Maysaloun Merhi, Afsheen Raza, Varghese Philipose Inchakalody, Abdulqadir Jeprel Japer Nashwan, Niloofar Allahverdi, Roopesh Krishnankutty, Shahab Uddin, Abdul Rehman Zar Gul, Mohammed Ussama Al Homsi, Said Dermime

**Affiliations:** ^1^National Center for Cancer Care and Research, Hamad Medical Corporation, Doha, Qatar; ^2^Translational Cancer Research Facility and Clinical Trial Unit, Interim Translational Research Institute, Hamad Medical Corporation, Doha, Qatar; ^3^Interim Translational Research Institute, Hamad Medical Corporation, Doha, Qatar

**Keywords:** head and neck squamous cell carcinoma, programmed cell death protein-1, nivolumab, NY-ESO-1 antibody, NY-ESO-1-specific T cells, cytokine profile

## Abstract

Targeting the programmed cell death protein-1 (PD-1)/PD-1 ligand (PD-L1) pathway has been shown to enhance T cell-mediated antitumor immunity. Clinical responses are limited to subgroups of patients. The search for biomarkers of response is a strategy to predict response and outcome of PD-1/PD-L1 checkpoint intervention. The NY-ESO-1 cancer testis antigen has been considered as a biomarker in head and neck squamous cell carcinoma (HNSCC) patients and can induce both specific NY-ESO-1 antibody and T cells responses. Here, we correlated clinical responsiveness to anti-PD-1 (nivolumab) treatment with immunity to NY-ESO-1 in a patient with recurrent HNSCC. The patient was treated with second-line treatment of nivolumab and had a stable disease for over 7 months. His NY-ESO-1 antibody was found to be lower after the third (****p < 0.0001) and the fifth (****p < 0.0001) cycles of treatment compared to base line, and this was in line with the stability of the disease. The NY-ESO-1-specific T cells response of the patient was found to be increased after the third and the fifth (**p = 0.002) cycles of treatment but had a significant decline after progression (**p = 0.0028). The PD-1 expression by the patient’s T cells was reduced 15-folds after nivolumab treatment and was uniquely restricted to the CD8^+^ T cells population. Several cytokines/chemokines involved in immune activation were upregulated after nivolumab treatment; two biomarkers were reduced at progression [interleukin (IL)-10: ****p < 0.0001 and CX3CL1: ****p < 0.0001]. On the other hand, some cytokines/chemokines contributing to immune inhibition were downregulated after nivolumab treatment; two biomarkers were increased at progression (IL-6: ****p < 0.0001 and IL-8: ****p < 0.0001). This data support the notion that the presence of anti-NY-ESO-1 integrated immunity and some cytokines/chemokines profile may potentially identify a response to PD-1 blockade in HNSCC patients.

## Introduction

Head and neck Squamous cell carcinoma (HNSCC) is the sixth most common cancer worldwide, accounting for approximately 6% of all cases and is responsible for an estimated 1–2% of all cancer deaths (1, 2). More than 90% of tumors in the head and neck are squamous cell carcinomas (3). The majority of HNSCC patients present with advanced-stage disease characterized by significant rates of local failure and distant metastases subsequent to radiotherapy (4, 5). Advances in surgery, chemotherapy, and radiotherapy have not altered the survival rates of patients with HNSCC over the past two decades (6). Emerging evidence supports an important role of the immune system in the development and evolution of HNSCC in which the status of the immune system is likely to be of prognostic value. It has been demonstrated that patients with HNSCC have either a downregulation of their antitumor immune responses and tumor progression or relapse that correlates with immune dysfunction (7). Immunotherapy has emerged as a promising treatment approach for cancer with extraordinary survival in selected patients. Immunotherapy using immune-modulating antibodies, which is based on reconstitution of the efficacy of pre-existing immune responses in patients, is used to help counteracting various tumor evasion strategies (8). Nivolumab is an immune-modulating antibody against the programmed cell death protein-1 (PD-1). PD-1 is an immune checkpoint receptor found on the surface of T cells that downregulates their activation (8, 9). Nivolumab has been recently approved by the FDA as an option for the second-line treatment of recurrent and/or metastatic HNSCC (10). The results from a very recent prospective randomized trial using this antibody heralded a new era of anti-cancer therapy in HNSCC (11, 12). Because such immune-modulating antibodies are known to *unleash the brake of the immune system* (13, 14), the presence of a pre-existing immune response is essential for the success of such therapy. Therefore, the identification of target antigens for such immune responses has become precedence. The NY-ESO-1 cancer testis antigen has been shown to be expressed in HNSCC patients and to exhibit the capacity to induce both natural antibody and T cell responses (15). Because of its high tumor-specificity and immunogenicity, the NY-ESO-1 antigen may represent an attractive target for specific immunotherapy of HNSCC. Indeed, it has been demonstrated that melanoma patients treated with ipilimumab had an increased rate of NY-ESO-1-specific immunity that was associated with improved clinical benefit of the treatment, especially in patients developing both NY-ESO-1-specific antibody and specific CD8^+^ T cells (16).

We therefore speculate that such pre-existing immunity to the NY-ESO-1 antigen should be enhanced after anti-PD-1 treatment leading to improved clinical benefit of the patient. We showed here that anti-PD-1 (nivolumab) treatment of an HNSCC patient modulated his immune response to the NY-ESO-1 antigen. We have also showed differential expression of important cytokines/chemokines markers that correlated with the patient clinical outcome.

## Case Report

A 71-year-old Qatari male patient was diagnosed with oral cavity HNSCC with stage cT4 N0 M0 in 1997 and underwent radiotherapy in London, UK. He developed post-radiation necrosis and neck fistula, which was treated with a skin flap. After initial chemo-radiation in 2016, a recurring HNSCC involving the supraglottic region and tongue base was identified. On the 12th of January 2017, a second-line treatment with nivolumab was started (3 mg/kg every 2 weeks for five cycles) after declining chemotherapy. However, due to non-compliance the patient refused further treatment. Two CT scans of the patient neck were taken before treatment and 10 days after the fifth cycle of the treatment. PET CT scan was carried out 239 days after the fifth cycle (7 months, 25 days) of treatment. The antibody response to the NY-ESO-1 antigen was measured in the plasma using enzyme-linked immunosorbent assay (ELISA) against a known immunogenic NY-ESO-1 peptide. The cellular response to the NY-ESO-1 antigen was investigated in patient’s peripheral blood mononuclear cells (PBMCs) using an enzyme-linked immunospot (ELISPOT) assay for interferon-gamma (IFN-γ) production by T cells against the NY-ESO-1 overlapping peptides. Flow cytometry was used to determine the expression of PD-1 in the patient CD3^+^ T cells before and after nivolumab treatment. A panel of 27 plasma biomarkers (cytokines and chemokines) was analyzed by multiplex analysis.

### Clinical Response to Nivolumab

**Figure 1:**
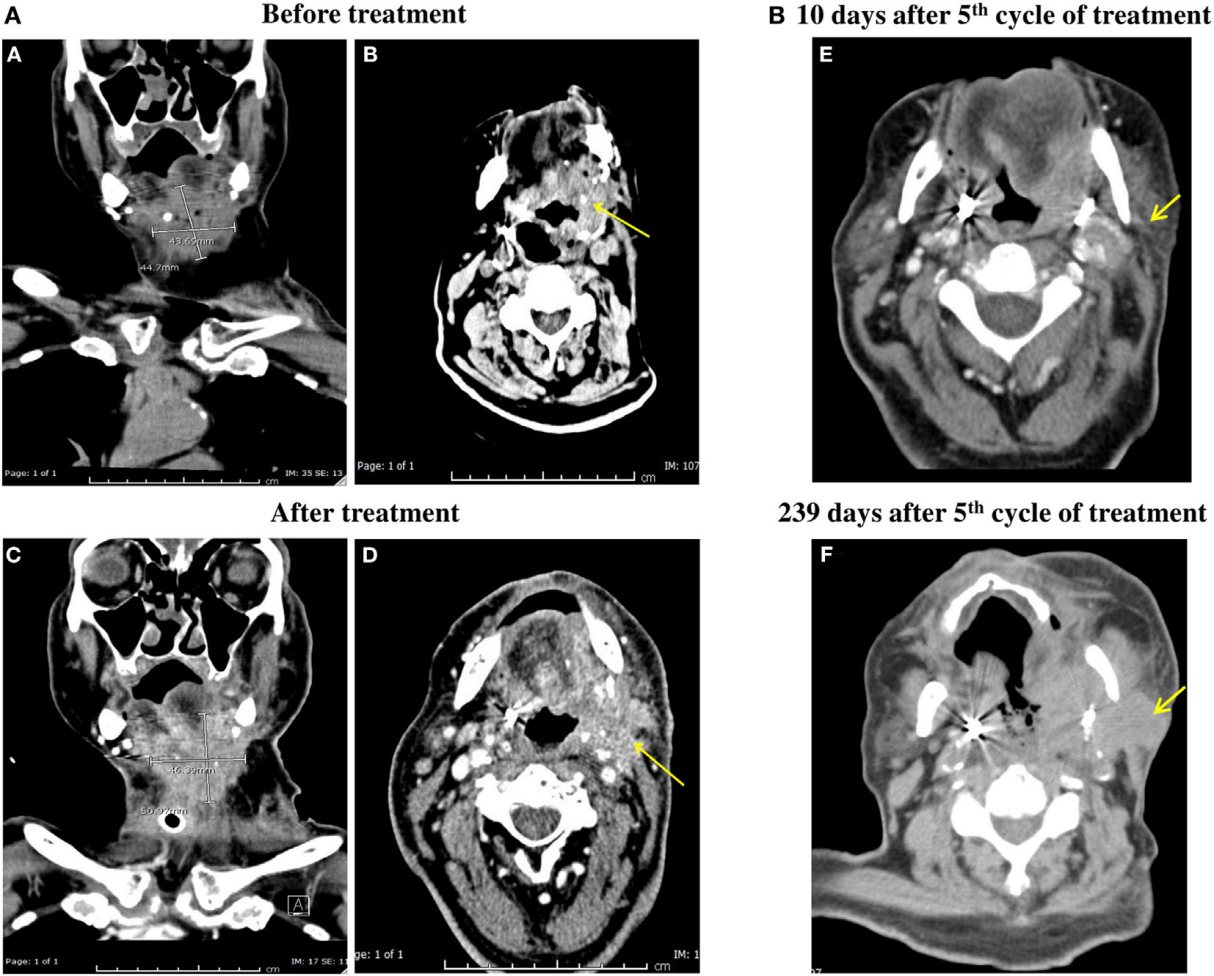
**(A)** CT scan of the patient neck with IV contrast. Irregular infiltrative mass in the left side of the neck adjacent to the base of the tongue, invading the oropharynx and extending caudally to supraglottic and glottic larynx was shown both before and after the fifth cycle of anti-programmed cell death protein-1 (PD-1) treatment [**(B,D)** respectively]. It shows mild increase in size measuring about 5.1 cm × 4.6 cm 10 days after the fifth cycle **(C)** compared to 4.5 cm × 4.3 cm before anti-PD-1 treatment **(A)**. **(B)** PET CT carried out at day 239 after fifth cycle (7 months, 25 days) of anti-PD-1 treatment showing progression of the disease **(F)** compared tp PET CT obtained at 10 days after the fifth cycle **(E)**.

After the fifth cycle of nivolumab treatment, the patient’s bleeding from the tumor site at the neck stopped and CT scan follow-up showed stable disease, no progression, or distant metastasis (Figure 1A). It showed a mild increase in size, measuring about 5.1 cm × 4.6 cm, 10 days after the fifth cycle (Figures 1A–C) compared to 4.5 cm × 4.3 cm before nivolumab treatment (Figures 1A–A) which suggests of pseudo-progression. On the other hand, 163 days (5 months, 10 days) after the fifth cycle of nivolumab treatment, the patient was seen by an oncologist and found to be in a fair general condition. Because the patient declined to have any follow-up CT scans and blood tests, a mobile medical team visited him several times and evaluated him as feeling well with an on and off cough. The patient also complained of limited pain on the left sub-mandibular angle but physical examination showed no palpable mass in that area. The patient was again seen by the medical team 194 days (6 months, 10 days), 209 days (6 months, 25 days), and 226 days (7 months, 12 days) after the fifth cycle of nivolumab treatment. On all visits, the patient had some cough with blood, a small soft tissue mass was observed on the left side of the neck. However, no hard mass was observed. The patient was admitted to the National Center for Cancer Care and Research (NCCCR) 234 days (7 months, 20 days) after the fifth cycle of nivolumab treatment with left mandibular pain and swelling. He had productive cough of copious whitish sputum with no fever. PET CT scan was carried out at day 239 after the fifth cycle (7 months, 25 days) and the patient was found to have progressed (Figures 1B–F).

### Determination of Antitumor Immune Response

**Figure 2:**
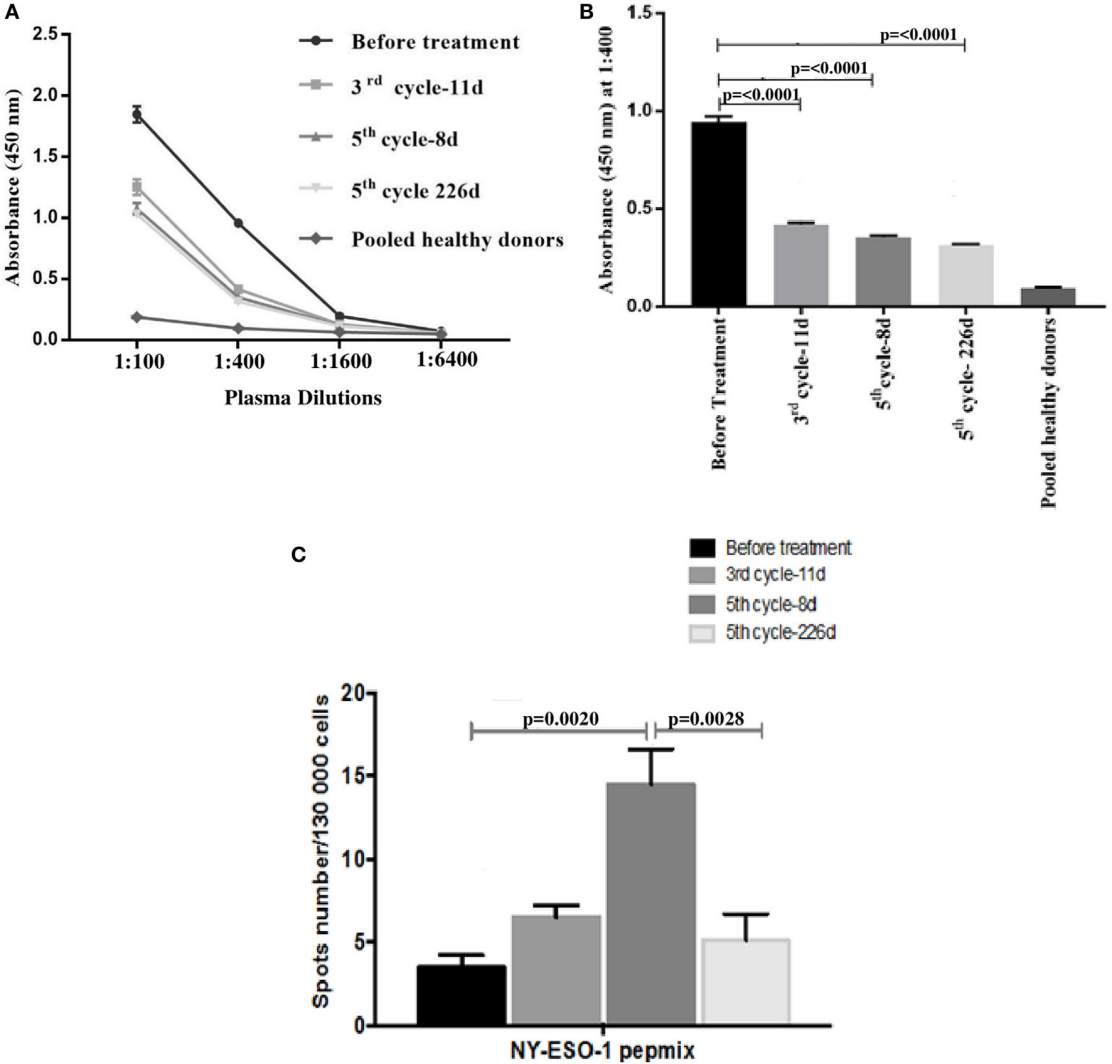
Antibody response to the NY-ESO-1 antigen as measured in the plasma by enzyme-linked immunosorbent assay (ELISA). **(A)** The results are expressed as the mean OD value and error bars indicate the SD for the triplicate values in each dilution. Out of the four different plasma dilutions tested (1:100, 1:400, 1:1,600, and 1:6,400), 1:100 and 1:400 were found to be the optimum dilutions to differentiate the anti-NY-ESO-1 antibody level before and after nivolumab treatment. **(B)** Bar graph represents the mean OD values were measured at 1:400 dilution. Each ELISA experiment was repeated six times and the shown data corresponds to one representative experiment. **(C)** Enzyme-linked immunospot (ELISPOT) assay for interferon-gamma production to investigate T cell response to the NY-ESO-1 antigen in patient’s peripheral blood mononuclear cells against NY-ESO-1 overlapping peptides (PepMix). The assay was repeated three times and the shown data corresponds to one representative experiment. Statistical analysis for ELISA and ELISPOT were performed using non-parametric unpaired ANOVA followed by multiple comparison Dunnet’s test and *p* values <0.05 were considered statistically significant.

The humoral immune response to the NY-ESO-1 antigen was measured. ELISA results showed that out of the four different plasma dilutions tested (1:100, 1:400, 1:1,600, and 1:6,400), 1:100 and 1:400 were found to be the optimum dilutions to differentiate the anti-NY-ESO-1 antibody level before and after nivolumab treatment (Figure 2A). ELISA results showed that the NY-ESO-1 antibody levels at 1:400 plasma dilution were significantly higher before nivolumab treatment compared to samples taken 11 days after the third cycle (third cycle-11 days, ****p < 0.0001), 8 days after the fifth cycle (fifth cycle-8 days, ****p < 0.0001), and at progression stage (fifth cycle-226 days, ****p < 0.0001) (Figure 2B). We used pooled plasma from five healthy donors as a negative control. Interestingly, the patient plasma recognized the NY-ESO-1 (11–30 amino acids) peptide which represents one of the most known immunogenic epitope of the NY-ESO-1 antibody. No response was obtained with the non-immunogenic long peptide (amino acids 85–111) (data not shown). The ELISA result was confirmed using Western Blot analysis which showed the recognition of an 18 kDa band by the NY-ESO-1 antibody in the patient plasma (data not shown).

The cellular immune response to the NY-ESO-1 antigen was measured in the PBMCs of the patient before and after nivolumab treatment for IFN-γ secretion using ELISpot assay. Specific IFN-γ secretion was demonstrated against a pool of 43 peptides representing the whole length of the NY-ESO-1 protein (PepMix) (Figure 2C). IFN-γ secretion was slightly increased in T cells tested 11 days after the third cycle (third cycle-11 days) and was significantly higher 8 days after the fifth cycle (fifth cycle-8 days, **p = 0.002) of nivolumab treatment compared to control before treatment. Interestingly, there was a significant decrease in IFN-γ secretion by the patient T cells collected at progression (fifth cycle-226 days, **p = 0.0028) (Figure 2C). No IFN-γ secretion was obtained in the presence of the negative control, PSA PepMix (data not shown).

**Figure 3:**
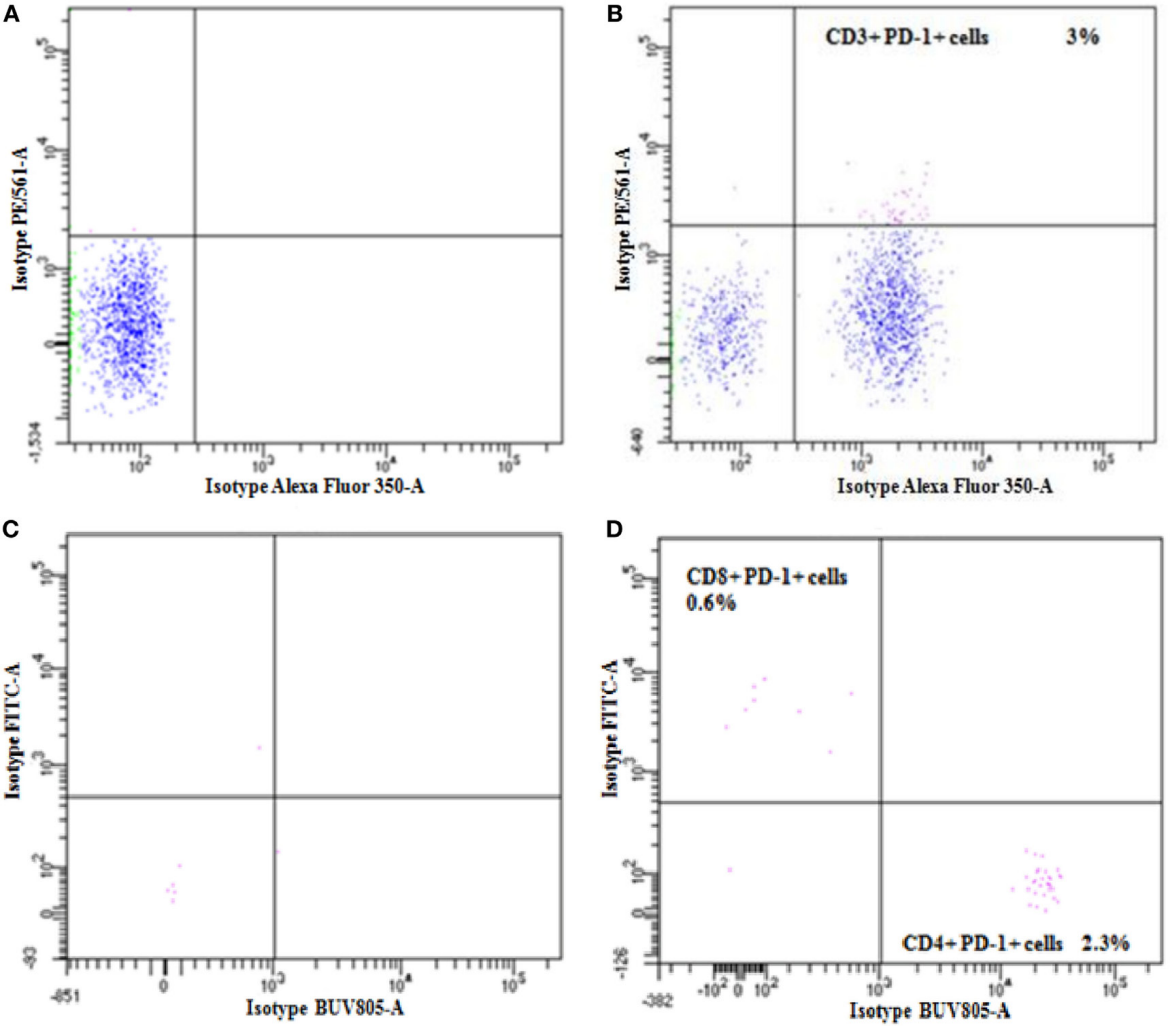
Flow cytometry was used to determine the expression of programmed cell death protein-1 (PD-1) in the patient CD3^+^, CD4^+^, and CD8^+^ T cells before nivolumab treatment. Panels **(A,B)** are dot plots for isotype control and for PD-1 staining in CD3^+^, respectively. Isotype control and PD-1 staining in CD4^+^ and CD8^+^ cells are represented in panels **(C,D)**.

The PD-1 expression by T cells was investigated in the PBMCs of the patient before and after the fifth (fifth cycle-8 days) cycle of nivolumab treatment using flow cytometry analysis. Nivolumab treatment demonstrated a 15-fold decrease in the expression of PD-1 by the CD3^+^ T cells when compared to the value obtained before treatment (Figure 3B). Although both subsets of CD4^+^ and CD8^+^ T cells expressed the PD-1 molecule, its expression was dominant in the CD4^+^ T cells population before treatment (Figure 3D). The expression of PD-1 in the CD4^+^ and CD8^+^ T cells population was below detection limits after nivolumab treatment (data not shown).

**Figure 4:**
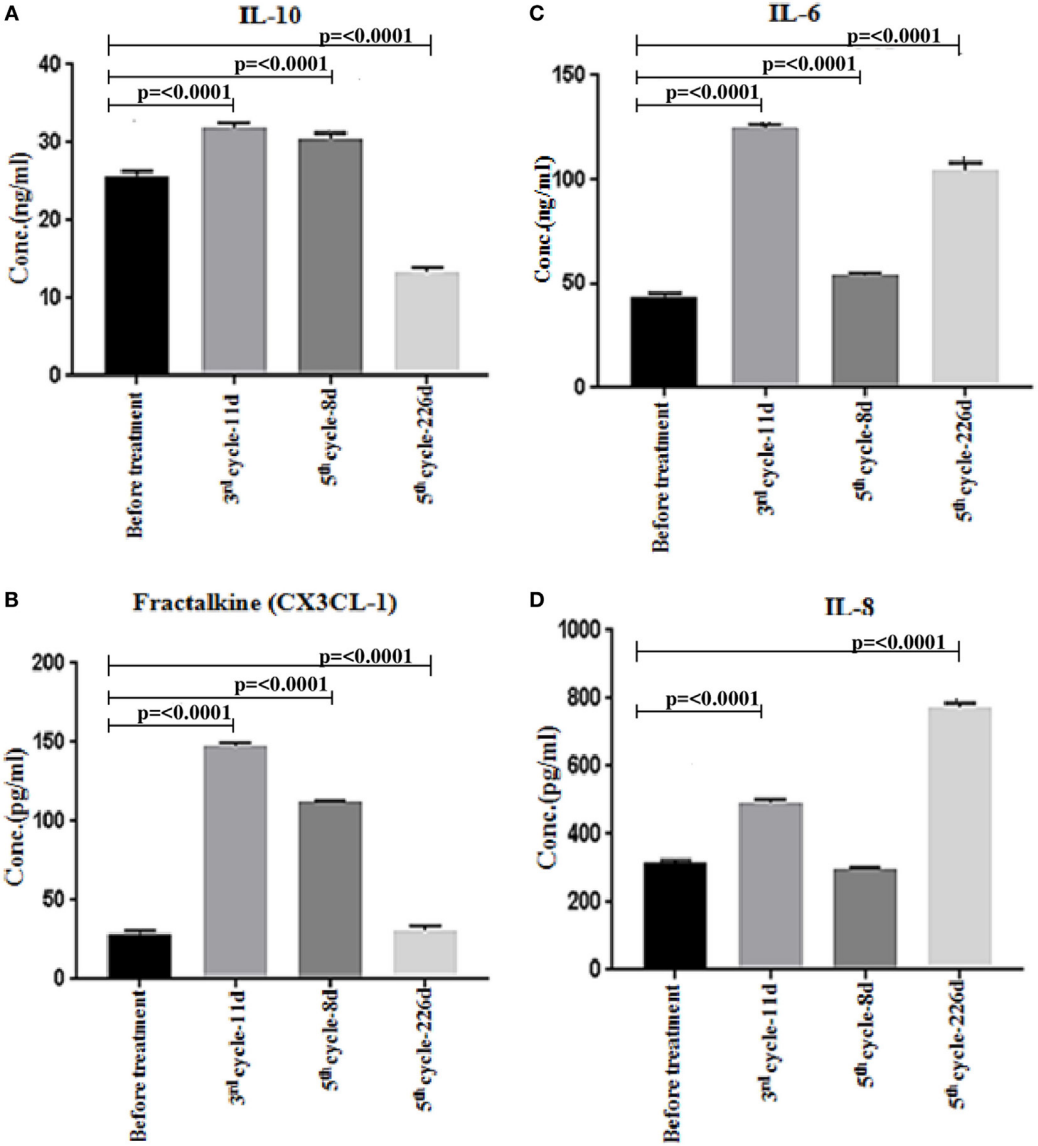
Multiplex analysis of cytokines/chemokines in patient plasma before and after nivolumab treatment, and after progression. **(A,B)** Significant downregulation of the immune activation biomarkers (IL-10 and CX3CL-1 also known as Fractalkine) at progression (fifth cycle-226 days). **(C,D)** Significant upregulation of the immune inhibition biomarkers (IL-6 and IL-8) at progression (fifth cycle-226 days). The assay was repeated three times and the shown data corresponds to one representative experiment. Statistical analysis was performed using non-parametric unpaired ANOVA followed by multiple comparison Dunnet’s test and *p* values <0.05 were considered statistically significant.

The cytokine/lymphokine profile was investigated in the plasma of the patient before and after the third cycle (third cycle-11 days) and the fifth cycle (fifth cycle-8 days) of nivolumab treatment as well as at progression (fifth cycle-226 days) using multiplex analysis. We have classified the cytokine/lymphokine profile, based on its upregulation or downregulation status after nivolumab treatment, into two groups (Tables 1 and 2). Group 1 comprises 10 biomarkers that were significantly upregulated after the third cycle-11 days (Table 1). These are IFN-γ, tumor necrosis factor-alpha (TNF-α), interleukin (IL)-6, IL-8, IL-10, granulocyte-macrophage colony-stimulating factor (GM-CSF), macrophage inflammatory protein-1β (MIP-1β), chemokine C-X3-C motif ligand 1 (CX3CL-1), CXCL-11, and soluble CD137 (sCD137). It is important to mention that four of the biomarkers (IL-10, GM-CSF, CX3CL-1, and sCD137) also continued to rise after the fifth cycle (fifth cycle-8 days) of nivolumab treatment (Table 1). Group 2 comprises five biomarkers that were significantly downregulated after the third cycle-11 days and also continued to decline after the fifth cycle (fifth cycle-8 days) of nivolumab treatment (Table 2). These are granzyme A, granzyme B, perforin, soluble first apoptosis signal (sFAS), and IL-17A. Two biomarkers (IL-10 and CX3CL-1 also known as Fractalkine), important for immune activation, were significantly reduced at progression (fifth cycle-226 days, Figures 4A,B). Moreover, two biomarkers (IL-6 and IL-8), important for immune inhibition, were significantly upregulated at progression (fifth cycle-226 days, Figures 4C,D). The remaining 12 biomarkers analyzed [IL-2, IL-4, IL-5, IL-7, IL-12 (p70), IL-13, IL-21, IL-23, MIP-1α, MIP-3α, MIP-1β, and sFASL] showed no significant change (data not shown).

**Table 1:** Plasma concentrations of upregulated cytokines/chemokines after nivolumab treatment.

Cytokines/chemokines	^a^Before treatment Conc. (ng/ml)	^a^After third cycle (third cycle-11 days) Conc. (ng/ml)	*p*-Value	^a^After fifth cycle (fifth cycle-8 days) Conc. (ng/ml)	*p*-Value
IFN-γ	0.014 ± 1.15	0.0356 ± 1.05	****0.0001	0.018 ± 1.12	****0.0001
TNF-α	0.156 ± 0.57	0.575 ± 0.61	****0.0001	0.152 ± 1.52	*NS*
IL-6	0.038 ± 0.75	0.171 ± 0.63	****0.0001	0.047 ± 1.5	****0.0001
IL-8	0.313 ± 1.20	0.709 ± 1.22	****0.0001	0.316 ± 1.10	**0.004
IL-10	0.0316 ± 1.01	0.076 ± 1.1	****0.0001	0.058 ± 1.04	****0.0001
GM-CSF	0.018 ± 0.57	0.022 ± 1.0	**0.003	0.028 ± 1.5	****0.0001
MIP-1β	0.108 ± 1.2	0.125 ± 1.6	****0.0001	0.110 ± 1.2	**0.007
CX3CL-1	0.068 ± 1.32	0.103 ± 1.02	****0.0001	0.096 ± 1.51	****0.0001
CXCL-11	1.885 ± 0.7	2.002 ± 0.8	****0.0001	1.889 ± 0.7	*NS*
sCD137	50.6 ± 057	65.5 ± 1.32	****0.0001	79.1 ± 1.39	****0.0001

*^a^Values represent concentration (mean ± SD)*.

*Statistical analysis was carried out using non-parametric unpaired ANOVA followed by Dunnet’s multiple comparison test*.

*p Value: <0.05 significant*.

**Indicates significant.

****Indicates highly significant.

*NS, not significant; Conc, concentration; ng/ml, nanogram per milliliter; IFN-γ, interferon-gamma; TNF, tumor necrosis factor-alpha; IL-6, interleukin-6; IL-8, interleukin-8; GM-CSF, granulocyte-macrophage colony-stimulating factor; MIP-1β, macrophage inflammatory protein-1β; CX3CL-1, chemokine C-X3-C motif ligand 1; CXCL-1, C-X-C motif chemokine 11; sCD137, soluble CD137*.

**Table 2:** Plasma concentrations of downregulated cytokines/chemokines after nivolumab treatment.

Cytokines/chemokines	^a^Before treatment Conc. (ng/ml)	^a^After third cycle (third cycle-11 days) Conc. (ng/ml)	*p*-Value	^a^After fifth cycle (fifth cycle-8 days) Conc. (ng/ml)	*p*-Value
Granzyme A	193.5 ± 0.12	21.6 ± 0.15	****0.0001	23.8 ± 0.28	****0.0001
Granzyme B	2,111 ± 1.05	1,186.8 ± 1.05	****0.0001	1183.2 ± 1.02	****0.0001
Perforin	9,367 ± 0.25	5,236 ± 1.04	****0.0001	6,483 ± 1.03	****0.0001
sFAS	135.6 ± 1.6	63.6 ± 1.05	****0.0001	115.6 ± 1.23	****0.0001
IL-17A	17.3 ± 0.57	9.6 ± 1.04	****0.0001	6.5 ± 1.02	****0.0001

*^a^Values represent concentration (mean ± SD)*.

*Statistical analysis was carried out using non-parametric unpaired ANOVA followed by Dunnet’s multiple comparison test*.

*p Value: <0.05 significant*.

****Indicates highly significant.

*Conc, concentration; ng/ml, nanogram per milliliter; IL-17A, interleukin-17A; sFAS, soluble first apoptosis signal*.

## Methods

### Sample Collection and PBMCs Isolation

Peripheral blood samples were obtained in lithium heparin tubes 12 days before nivolumab treatment, 11 days after the third cycle, then 8 and 226 days after the fifth cycles of nivolumab treatment. Plasma was obtained after centrifugation of the blood at 1,200 rpm, frozen at −85°C and used subsequently for ELISA and multiplexing assays. PBMCs were isolated by density gradient centrifugation using Ficoll^®^ Paque Plus (GE Healthcare) and SepMate™ tubes (STEM Cells technologies) according to the manufacturer’s instructions. The obtained PBMCs were frozen at −150°C and used in ELISpot assay.

### Enzyme-Linked Immunosorbent Assay

96-well microtiter plates (Thermo Scientific) were coated with bicarbonate/carbonate buffer containing 10 µg/ml of the NY-ESO-1 peptide spanning its region of 11–30 amino acids (JPT Peptide Technologies GmbH, Germany) representing the most immunogenic epitope of the NY-ESO-1 antibody (17). We used a non-immunogenic long peptide (amino acids 85–111) from NY-ESO-1 as a negative control. After overnight incubation at 4°C, plates were washed with PBS containing 0.05% Tween-20 (PBS-T) (Sigma Aldrich) and blocked for 2 h at room temperature (RT) with PBS containing 5% skimmed milk powder (Sigma Aldrich). The Plasma was incubated for 2 h at RT at different dilutions, 1:100, 1:400, 1:1,600, and 1:6,400, in PBS containing 5% skimmed milk powder. Plates were washed with PBS-T and incubated for 1 h at RT with HRP-conjugated goat-anti-human antibody (Abcam) diluted 1:4,000 in PBS containing 5% skimmed milk powder, followed by measurement of the HRPO activity using 3,3′,5,5′-Tetramethylbenzidine (TMB) substrate solution (Sigma Aldrich) at 450 nm. The NY-ESO-1 antibody was considered positive if the mean OD value of the 1:100 dilution of the plasma is higher than the mean OD value of the healthy donor plus three times the SD (18).

### Western Blot Analysis

The anti-NY-ESO-1 antibody response were tested in the plasma by a standard western blot analysis using 1 µg of recombinant NY-ESO-1 protein and plasma at 1:100, 1:500, 1:1,000, 1:2,000, and 1:4,000 dilutions. HRPO-conjugated goat-anti-human antibody (Abcam) diluted at 1:4,000 was used as secondary reagent. The protein band of NY-ESO-1 was detected using chemiluminescence (19). Western blot bands from three independent experiments were quantified using the software Image J (http://rsb.info.nih.gov/ij/) (20).

### ELISpot Assay

Enzyme-linked immunospot assay was performed to enumerate the patient NY-ESO-1-specific T cells secreting IFN-γ after an *in vitro* re-challenge with the NY-ESO-1 antigen. A pool of 43 Peptides (15mers with 11 aa overlap) representing the whole length of the NY-ESO-1 protein (PepMix™ Human NY-ESO-1, JPT Peptide Technologies) were used. The human IFN-γ ELISpotPLUS kit (Mabtech) was used according to the manufacturer’s protocols. PBMCs were plated in duplicates at 130,000 cells/well/200 μl in complete RPMI media (Life Technologies) supplemented with 10% heat inactivated fetal bovine serum (Life Technologies), 1% Penicillin and Streptomycin cocktail antibiotics (Life Technologies) and 1% GlutaMAX (Life Technologies). The NY-ESO-1 PepMix was added at 1 µg/ml. Anti-CD3 mAb (Mabtech) was used as a positive control. We used the PepMix pool for the prostate-specific antigen (PM-PSA, from JPT technologies) as negative control. After 48 h of incubation, plates were developed and analyzed using an automated ELISPOT reader (AID, Strasberg, Germany). The frequency of NY-ESO-1-specific T cells was expressed as specific spot forming unit (SFC) per input cell numbers.

### Flow Cytometry Analysis

Flow cytometry analysis was carried out to determine PD-1 expression by the patient T cells before and after nivolumab treatment. Cryopreserved PBMCs were thawed, washed, and suspended in cold PBS (Gibco). The cells were stained with CD3-BUV395 (BD Biosciences), CD4-BUV805 (BD Bioscience), CD8-FITC (eBioscience), and PD-1-PE (eBioscience) antibodies. Isotype controls corresponding all the tested antibodies were used. After 30 min incubation at RT, the stained cells were washed then acquired on BD FACSCalibur flow cytometer and the data was analyzed using FACSDiva 8.0.1 software (BD Biosciences).

### Multiplex Analysis

The quantification of the patient cytokines and chemokines (granzyme A, granzyme B, perforin, MIP-1 α, MIP-1 β, MIP-3α, ITAC, sFASL, sFAS, sCD137, GM-CSF, IFN-γ, TNF-α, IL-1 β, IL-2, IL-4, IL-5, IL-6, IL-7, IL-8, IL-10, IL-12, IL-13, IL-17A, IL-21, and IL-23) was performed using the human Cytokine/Chemokine Magnetic Bead multiplex assay (CD8^+^ T cell magnetic Bead panel and Human high sensitivity T cell magnetic bead panel kits) (MILLIPLEX MAP, EMD Millipore, Billerica, MA, USA) according to the manufacturer’s instructions. Fluorescent signals generated were detected using the multiplex array reader Bio-Plex 200 System (Bio-Rad Laboratories, Inc.). Briefly, raw data were initially measured as relative fluorescence intensity then converted to cytokine concentrations based on the standard curve generated from the reference concentrations supplied with the kit. All samples and standards were performed in duplicates and data were analyzed as mean of duplicates.

### Statistical Analysis

For ELISpot analysis, Multiplex analysis and ELISA a non-parametric unpaired ANOVA followed by multiple comparisons Dunnet’s test were used. Statistical analysis was performed using Graph Pad Prism 7.0 and *p* values <0.05 were considered statistically significant.

## Discussion

It has been recently demonstrated that patients with HNSCC have downregulation of their antitumor immune responses, and tumor progression or relapse correlates with immune dysfunction (7). PD-1 is an immune checkpoint receptor that is expressed mainly on T cells and limits T cell effector functions within the tumor site (8, 9). Furthermore, tumor cells can upregulate the expression of the PD-1 ligand (PD-L1) and further block the antitumor immune response (21). In this study, it has been shown that PD-L1 was expressed in 50–60% of HNSCC patients (21). Interestingly, blocking the PD-1/PD-L1 interaction with anti-PD-1 antibodies was reported to improve survival in recurrent HNSCC patients (9, 22). Indeed, treatment of recurrent HNSCC patients with nivolumab, in a recent phase III CheckMate 141 clinical trial, resulted in longer overall survival compared to treatment with a standard single agent therapy (11, 12). The trial was stopped early due to this survival advantage (11, 12). Based on this indication, nivolumab was recently approved to treat recurrent HNSCC patients (10). In the present report, nivolumab was used to treat a 71-year-old male patient with a long and recurrent history of HNSCC. It has been reported that pseudo-progression with an initial increase in total tumor burden; stable disease state with a slow steady decline in total tumor volume; or a presence of new lesions, were all associated with patients responding to anti-CTLA-4 treatment and were linked to favorable survival (23). The patient reported in our study had an initial increase in total tumor burden and displayed a stable disease with slow steady decline in total tumor volume over 7 months demonstrating an initial pseudo-progression phenomenon. The patient progressed after 7 months of nivolumab treatment. Although immunotherapy has advocated to continue after the disease first progression provided that the general condition of the patient had improved, our patient refused to continue nivolumab treatment after the fifth cycle.

It has been demonstrated that changes in NY-ESO-1 antibody levels correlate with the evolution of NY-ESO-1 positive disease and tend to disappear with tumor resection or therapy-induced regression (24). Furthermore, the humoral immune response against the NY-ESO-1 antigen is frequently observed in patients with NY-ESO-1 expressing tumors and no NY-ESO-1 antibody has been detected in healthy controls and/or patients with NY-ESO-1 negative tumors (24). Plasma samples collected from the patient before nivolumab treatment expressed significant levels of NY-ESO-1 antibody compared to a pool of five plasma samples collected from healthy controls. This confirms the presence of NY-ESO-1 antigen in the patient’s tumor, as the induction and maintenance of NY-ESO-1 antibody was shown to be dependent on the presence of NY-ESO-1 expressing tumors. Moreover, we have shown in this report significant reduction in NY-ESO-1 antibody levels after the third (third cycle-11 days) and fifth (fifth cycle-8 days) cycles of nivolumab treatment compared to levels obtained before treatment. We have also demonstrated that the patient at progression stage (fifth cycle-226 days) did not have elevated NY-ESO-1 antibody levels raising the possibility of immune selection of NY-ESO-1 antigen negative variants (25).

We have shown that the patient’s T cells response to the NY-ESO-1 antigen was slightly increased after the third cycle (third cycle-11 days) and was significantly higher at the fifth (fifth cycle-8 days) cycle of nivolumab treatment. This is in line with another study demonstrating that treatment with an anti-PD-1 antibody increased the NY-ESO-1-specific T cells expansion in melanoma patients (26). Moreover, it has been demonstrated that T cells response to NY-ESO-1 antigen correlates with the patients’ clinical benefit in melanoma treated with anti-CTLA-4 antibody (16). Our data also showed that the T cells response detected after anti-PD-1 treatment was significantly declined after the stage of progression (fifth cycle-226 days). It has been shown recently that the expression of PD-1 on CD4^+^ T cells has a prognostic value in NSCLC patients, as high expression of this molecule was associated with a shorter progression-free survival/overall survival (27). In line with this, we showed here that the majority of all PD-1^+^/CD3^+^ T cells analyzed before nivolumab treatment were of the CD4^+^ T cells population (Figure 3). Treatment with an anti-PD-1 antibody was shown to increase NY-ESO-1-specific CD8^+^ T cells expansion in melanoma patients and in contrast to EBV, influenza, or Melan-A/MART-1-specific CD8^+^ T cells, NY-ESO-1-specific CD8^+^ T cells uniquely express the PD-1 molecule (26). In line with this, nivolumab treatment may expand NY-ESO-1-specific CD8^+^ T population expressing the PD-1 molecule in our patient (Figures 3A–D).

It has been previously demonstrated that in addition to the direct tumor lytic activity of CD8^+^ T cells, CD4^+^ T cells provide a protective function by cytokines secretion and inflammatory reactions. Our data showed that both Th_1_ and Th_2_ T cells are involved in the immune response of the patient and this was correlated with the cytokines/lymphokines profile produced upon response to nivolumab treatment (Figure 4). We have shown that several cytokines/chemokines, important for immune activation were upregulated after nivolumab treatment in which one important cytokine (IL-10) and chemokine (CX3CL1/Fractalkine) were significantly reduced at tumor progression. IL-10 has been known as an anti-inflammatory cytokine produced primarily by antigen-presenting cells, which exerts negative regulatory effects on pro-inflammatory cytokines by downregulating their synthesis (28). However, it has been shown recently that IL-10 plays also a major role as an immune-activating cytokine in cancer immunotherapy (29). In this respect, IL-10 induces production of IFN-γ that strongly induces the expression of MHC and costimulatory molecules, therefore both IL-10 and IFN-γ stimulate TCR signaling enabling T cells activation and proliferation (29). Interestingly, it has been recently demonstrated in advanced melanoma that IL-10 levels in nivolumab responders were significantly higher than those in the non-responders (30) supporting our current findings. CX3CL1 is a chemokine induced by inflammatory cytokines such as TNFα, IL-1β, and IFN-γ and its role is to recruit immune cells at tumor sites and to boost antitumor immune responses (31). Indeed, high expression of the CX3CL1 molecule by tumor cells was found to correlate with a good prognosis and with increased tumor-infiltrating CD8^+^ T cells, natural killer cells, and dendritic cells in breast carcinoma (31). It has been demonstrated recently that high expression of CX3CL1 in colorectal cancer was significantly associated with more favorable patients’ prognosis whereas low expression identifies a subset of patients with significantly higher risk of developing distant metastasis and rapid tumor progression (32). Furthermore, a recent study has shown that CX3CR1, a receptor for CX3CL1, is exclusively expressed in a subset of CD8^+^ effector memory T cells infiltrating tumor tissues in patients responding to anti-PD-1 therapy. This study has also demonstrated that PD-1 is mainly expressed in such T cells population (33). This is in line with our data showing the significant induction of CX3CL1 after nivolumab treatment and its downregulation at tumor progression (Figure 4B).

We have also shown that some cytokines/chemokines contributing to immune response inhibition were downregulated after nivolumab treatment in which one important cytokine (IL-6) and chemokine (IL-8) were significantly increased at progression. IL-6 is a pleiotropic cytokine that plays an important role in cell proliferation, survival, differentiation, migration, and invasion. It regulates tumor progression and tumor metastasis by modulating tumor angiogenesis and tumor lympho-angiogenesis (34). It has been demonstrated recently that IL-6 levels were markedly upregulated in HNSCC patients and high IL-6 expression independently predicts tumor recurrence, metastasis, and poor survival (34). We have shown here that IL-6 levels were significantly upregulated at both pseudo-progression and progression stages (Figure 4C). IL-8 is a chemokine secreted by malignant cells and tumor stroma cells across many different tumor types (35). It has been shown very recently that melanoma and NSCLC patients treated with anti-PD-1 antibody illustrated an early decrease in the levels of serum IL-8 and this was associated with longer overall survival (35). Moreover, high serum IL-8 levels in cancer patients presenting pseudo-progression also reflected true response to anti-PD-1 antibody treatment (35). We have shown in this report that IL-8 levels were significantly upregulated at both pseudo-progression and progression (Figure 4D). Finally, the significant low levels of cytolytic factors such as granzymes A and B, perforin, and sFAS in serum may be explained by the fact that activated T cells must migrate to come into contact with the tumor and after recognition of antigens, they release such cytolytic enzymes which recruit other cells of the immune system to destroy the tumor.

## Concluding Remarks

We have analyzed the expression of immunological markers before and after anti-PD-1 treatment in a patient with a long recurrent history of HNSCC and spontaneous immunity to the NY-ESO-1 antigen. The patient showed a transient regression and stability of the tumor after anti-PD-1 treatment for a period of seven and half months. The analysis of immunological markers in this patient showed a differential expression before and after anti-PD-1 treatment and at progression. Although we recognize one drawback of including only one patient in this current study, we suggest that this immunological monitoring would help in providing a critical understanding of the predictive value of NY-ESO-1 antibody, NY-ESO-1-specific T cells response, and the cytokines/chemokines cascade as biomarkers of the response to anti-PD-1 treatment. Further studies are needed to be performed in a larger number of cases from HNSCC patients treated with anti-PD-1 therapy to confirm our findings.

## Ethics Statement

The report was approved by the ethical board of the Hamad Medical Corporation, Doha, Qatar. The patient gave informed consent to carry out the laboratory analysis and to publish this report.

## Author Contributions

SD designed and supervised the study; SD, MM, AR, VI, RK, and SU performed research; MH, NA, AN, and AG collected clinical data; and SD wrote the paper. MM, AR, and VI contributed equally in writing sections of the manuscript. All authors contributed to manuscript revision, read and approved the submitted version.

## Conflict of Interest Statement

We declare that the research was conducted in the absence of any commercial or financial relationships that could be construed as a potential conflict of interest.
